# Enhancement in thermoelectric properties due to Ag nanoparticles incorporated in Bi_2_Te_3_ matrix

**DOI:** 10.3762/bjnano.10.63

**Published:** 2019-03-04

**Authors:** Srashti Gupta, Dinesh Chandra Agarwal, Bathula Sivaiah, Sankarakumar Amrithpandian, Kandasami Asokan, Ajay Dhar, Binaya Kumar Panigrahi, Devesh Kumar Avasthi, Vinay Gupta

**Affiliations:** 1 Department of Physics and Astrophysics, University of Delhi, New Delhi-110007, India; 2 Department of Physics, Sant Longowal Institute of Engg and Tech. Longowal, Punjab-148106, India; 3 Physics of Energy Harvesting Division, CSIR - National Physical Laboratory, Delhi-110007, India; 4 Materials Physics Division, Indira Gandhi Centre for Atomic Research, Kalpakkam-603102, India; 5 Material Science, Inter University Accelerator Centre, New Delhi, Delhi 110067, India; 6 Amity Institute of Nanotechnology, Amity University, Noida-Uttar Pradesh-201303, India

**Keywords:** bismuth telluride, nanoparticles, power factor, thermoelectric power

## Abstract

The present study aims to see the enhancement in thermoelectric properties of bismuth telluride (Bi_2_Te_3_) annealed at different temperatures (573 and 773 K) through silver (Ag) nano-inclusions (0, 2, 5, 10, 15 and 20 wt %). Transmission electron microscopy (TEM) images of Ag incorporated in Bi_2_Te_3_ annealed at 573 K shows tubular, pentagonal, trigonal, circular and hexagonal nanoparticles with sizes of 6–25 nm (for 5 wt % Ag ) and 7–30 nm (for 20 wt % Ag). Ag incorporated in Bi_2_Te_3_ annealed at 773 K shows mainly hexagonally shaped structures with particle sizes of 2–20 nm and 40–80 nm (for 5 wt % Ag) and 10–60 nm (for 20 wt % Ag). Interestingly, the samples annealed at 573 K show the highest Seebeck coefficient (*S*, also called thermopower) at room temperature (p-type behavior) for 5% Ag which is increased ca. five-fold in comparison to Ag-free Bi_2_Te_3_, whereas for samples with the same content (5% Ag) annealed at 773 K the increment in thermopower is only about three-fold with a 6.9-fold enhancement of the power factor (*S*^2^σ). The effect of size and shape of the nanoparticles on thermoelectric properties can be understood on the basis of a carrier-filtering effect that results in an increase in thermopower along with a control over the reduction in electrical conductivity to maintain a high power factor yielding a high figure of merit.

## Introduction

Bismuth telluride (Bi_2_Te_3_) is an important semiconductor widely used as thermoelectric (TE) material for room-temperature applications to convert waste heat into electricity. The efficiency of a TE material can be defined by figure of merit (ZT = *S*^2^σ*T*/*k*) and to enhance figure of merit (ZT), one needs to increase the power factor (*S*^2^σ, where *S* is the Seebeck coefficient or thermopower, σ is the electrical conductivity) or to decrease thermal conductivity (*k*). In bulk, all three parameters (*S*, σ, *k*) are interdependent. In bulk Bi_2_Te_3_, ZT is close to unity, it can be improved further with the introduction of nanostructures. The electron and phonon energy spectra can be controlled by tuning the size of nanostructures, which opens up new ways for ZT enhancement [1–2]. Since the introduction of nanotechnology in TE in 1993, various methods have been tried and it remained a challenge to fabricate highly efficient low-cost TE devices of nanostructured Bi_2_Te_3_ for industrial and daily life applications [1–7]. Bi_2_Te_3_ and its derivatives with Sb (p-type) and Se (n-type) are the best known commercial materials for room-temperature applications but these materials are not very efficient because of their low ZT [8–9].

To increase ZT, nanostructures play an important role in the simultaneous increase in power factor and reduction in phonon thermal conductivity (*k*_ph_) [10]. Recently, Faleev et al. [11] and Zebardaji et al. [12] performed theoretical calculations for the introduction of metal nano-inclusions in TE materials. This theory predicts the band bending at the metal–semiconductor interface will allow for the transmission of high energy electrons along with a blocking of low energy electrons. This electron energy filter results in enhancement of the Seebeck coefficient for a given carrier concentration. Several groups have used this approach using different metal–semiconductor combinations to improve thermoelectric properties [13–14]. One group has reported the synthesis of bismuth metal nanoparticles (NPs) were through a solvothermal method and the incorporation in Bi_2_Te_3_ synthesized by ball milling, which yielded a significant enhancement in power factor and ZT in Bi/Bi_2_Te_3_ due to the scattering of low-energy electrons by a barrier potential at the Bi–Bi_2_Te_3_ interface [15].

Improvement in TE properties has also been observed after the uniform dispersion of carbon nanotubes (CNTs) in Bi_2_Te_3_ [16]. Another group has also reported an enhancement of the Seebeck coefficient (*S*) in CNT/Bi_2_Te_3_ to 132 µV/K at 423 K [17]. In a recent report, a power factor of 43 µW·cm^−1^·K^−2^ for CuI-doped Bi_2_Te_3_ has been shown, which is higher than that of undoped Bi_2_Te_3_ and Cu -doped Bi_2_Te_3_ [18]. There are reports on Ag incorporation in Bi_2_Te_3_ especially for the preparation of 1D/3D-structured AgNWs/Bi_2_Te_3_ nanocomposites with enhanced thermoelectric properties, about 343% higher than that of pure Bi_2_Te_3_ [19]. Recently, Ag nanoparticles in a Bi_2_Te_3_ matrix have been synthesized and a dramatic enhancement in ZT by 304% for 2 vol % Ag in Bi_2_Te_3_ has been reported [20]. One research group submitted a patent on solvothermally synthesized low-dimensional nanocrystals of bismuth telluride covered by 0.001 wt %, 0.05 wt % Au or 0.05 wt % Ag metallic nanoparticles that yield both a higher power factor and figure of merit than pure Bi_2_Te_3_ [21].

An enhanced Seebeck coefficient (*S*) could compensate for the reduction in electrical conductivity to some extent maintaining *S*^2^σ as unaffected as possible. Through the metal fraction in the matrix or the synthesis conditions, one can control *S*, σ, or *k* independently. The above approaches showed issues regarding high cost and complicated syntheses that were difficult to repeat and unsuitable for scale-up. The enhancement in TE properties through Ag metal in a Bi_2_Te_3_ matrix motivated us to work on a detailed investigation of Ag in a Bi_2_Te_3_ matrix while focusing on an easy, reproducible low-cost synthesis suitable for scale-up. We show how to tune *S* and σ independently to achieve high *S*^2^σ values. One objective is to see which fraction of metal nanoparticles is required for the TE enhancement of a Bi_2_Te_3_ matrix and how different annealing temperatures lead to the different sizes and shapes of nanoparticles with different influence on thermoelectric properties. We have used different amounts of Ag microparticles (0, 2. 5, 10, 15 and 20 wt %) uniformly mixed with commercially purchased Bi_2_Te_3_, which was annealed at different temperatures (573 and 773 K)_._

## Experimental

Bismuth telluride (Bi_2_Te_3_) powder was purchased from Alfa Aesar (99.99% purity). Silver (Ag) powder (7–15 µm particles) was purchased from Ted Pella Inc. Different fractions (0, 2, 5, 10, 15 and 20 wt %) of Ag powder were added to Bi_2_Te_3_. The powder mixtures were well grinded and pressed to pellets. These pellets were then annealed at 573 or 773 K for 1 h under Ar atmosphere. Annealing temperatures were chosen to be below the melting point of Bi_2_Te_3_ (586 °C) and above the melting points of Bi (271 °C) and Te (449 °C). These samples were characterized by X-ray diffraction (XRD), transmission electron microscopy (TEM), and thermoelectric measurements. XRD measurements were performed using a Bruker D8 Avance diffractometer with Cu Kα (1.5406 Å) radiation. TEM investigations were carried out using a LIBRA 200 FE HRTEM. Gatan software [22] was used for analysis of HRTEM images of samples. Scanning electron microscopy with energy-dispersive spectroscopy (SEM EDS) was performed using a field-emission scanning electron microscope (FE-SEM) [MIRA\\, TESCAN]. Temperature-dependent thermoelectric measurements were carried out for all samples with size using a commercial instrument (Ulvac, ZEM3). The instrument error during TE measurements is ±5%.

## Results and Discussion

### X-ray diffraction and transmission electron microscopy selected area electron diffraction

**Figure 1:**
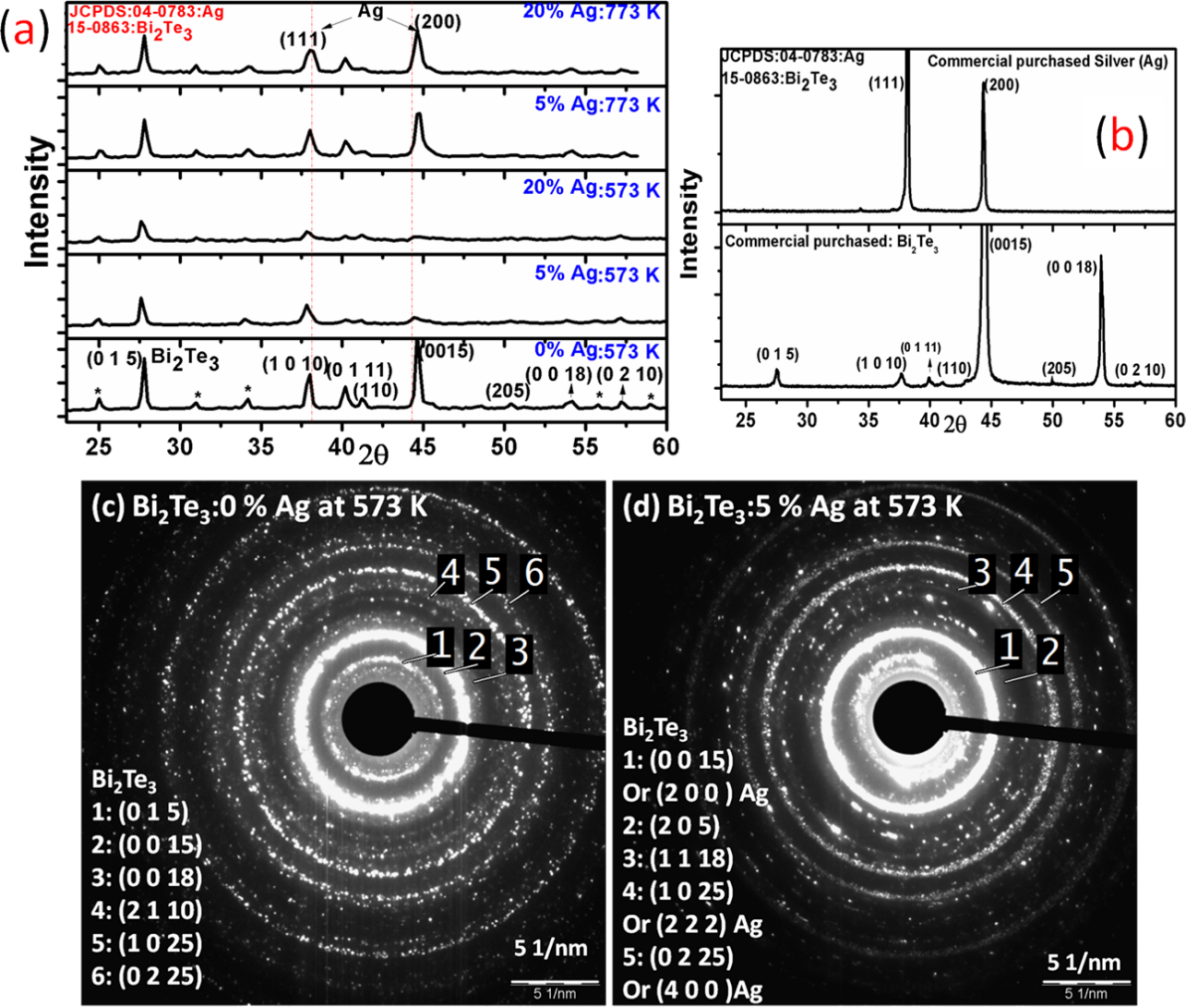
(a) XRD of Bi_2_Te_3_ with different concentration of Ag (0, 5, 20%) for annealing temperature 573 K, 773 K, (b) XRD of commercial purchased Ag, Bi_2_Te_3_ powder without annealing. SAED pattern of (c) Bi_2_Te_3_ and (d) Ag (5%) for annealing temperature 573 K.

X-ray diffraction (XRD) patterns of Bi_2_Te_3_ with different concentrations of Ag (0, 5 and 20 wt %) annealed at 573 and 773 K are shown in Figure 1a. The XRD patterns show main peaks of Bi_2_Te_3_ (rhombohedral) at 2θ = 27.8° (*d*-spacing: ≈0.321 nm), 37.98° (≈ 0.237 nm), 40.2° (≈0.223 nm), 41.2° (≈0.219 nm), 44.58° (≈0.203 nm), 50.4° (≈0.181 nm), 54.12° (≈0.169 nm) and 57.2° (≈0.161 nm) corresponding to the (0 1 5), (1 0 10), (0 1 11), (1 1 0), (0 0 15), (2 0 5), (0 0 18) and (0 2 10) planes, respectively (JCPDS 15-0863). There are some peaks marked with (*), which are speculated to be Bi_2_TeO_5_ (orthorhombic) for 2θ = 30.97° (≈0.289 nm) and 55.79° (≈0.166 nm) corresponding to the (0 0 4) and (9 1 2) planes, respectively (JCPDS 38-0420). At Ag concentrations of 5% and 20% in Bi_2_Te_3_ after 773 K annealing, all peaks are at the same positions. In samples with 5 and 20% Ag, annealed at 573 K, two peaks at 2θ = 37.98° and 44.58° matching the (1 1 1) and (2 0 0) planes of cubic with Ag (JCPDS-04-0783), respectively, can be seen. Bi_2_Te_3_ and Ag planes are in good agreement with the literature [20]. Figure 1b shows the XRD of commercially purchased Bi_2_Te_3_ and Ag powder without annealing.

The XRD results have also been verified by transmission electron microscopy selected area electron diffraction (TEM SAED) patterns of Bi_2_Te_3_ as shown in Figure 1c (0% Ag) and Figure 1d (5% Ag) annealed at 573 K. The planes in the SAED patterns were assigned using the Gatan software [22]. In Figure 1c, Bi_2_Te_3_ with 0% Ag, the identified planes for Bi_2_Te_3_ were (0 1 5), (0 0 15), (0 0 18), (2 1 10), (1 0 25) and (0 2 25). In Figure 1d the identified planes for Bi_2_Te_3_ were (from 1 to 5): (0 0 15), (2 0 5), (1 1 18), (1 0 25) and (0 2 25) respectively, whereas few planes were also assigned to Ag, i.e., ring 1: (2 0 0), ring 4: (2 2 2), ring 5: (4 0 0).SAED patterns of other samples, such as 20% Ag annealed at 573 K, and 5% and 20% Ag annealed at 773 K show the same plane indexing with negligible differences.

### Scanning electron microscopy with energy dispersive spectroscopy (SEM EDS)

**Figure 2:**
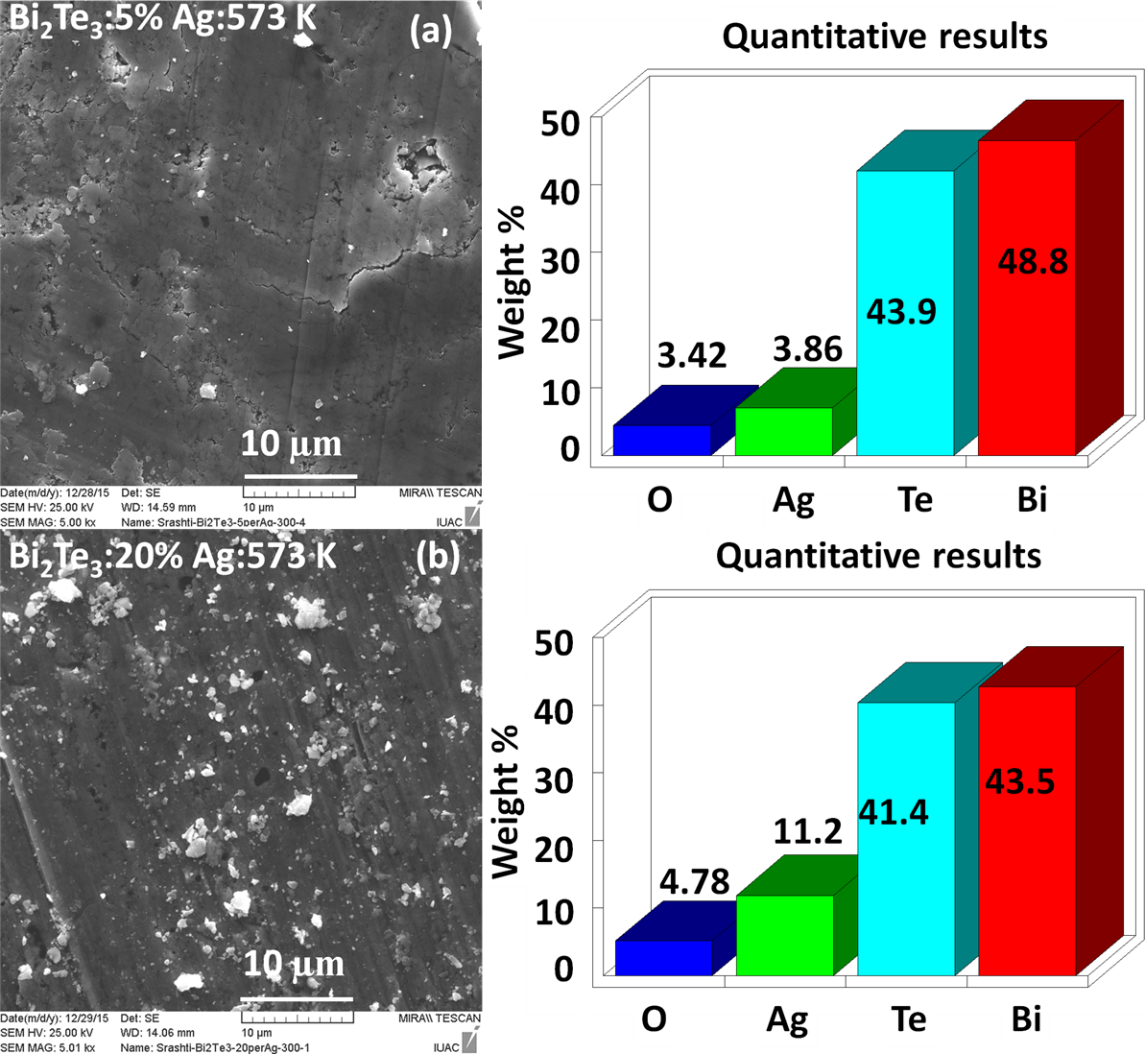
SEM and SEMEDS images of Bi_2_Te_3_ with (a) for 5% Ag, (b) for 20% Ag annealed at 573 K.

SEM EDS measurements of Bi_2_Te_3_:Ag (5% and 20% Ag) annealed at 573 K are shown in Figure 2a and Figure 2b, respectively. The values are averages of measurements at different locations. Ag, Bi and Te are present throughout the samples. The presence of oxygen cannot be neglected in the samples as its composition varies from 3.4% to 4.78% with an increase in Ag from 5% to 20%. From the SEM images and SEM EDS it is observed that Ag is well mixed with Bi_2_Te_3_.

### High-resolution transmission electron microscopy

**Figure 3:**
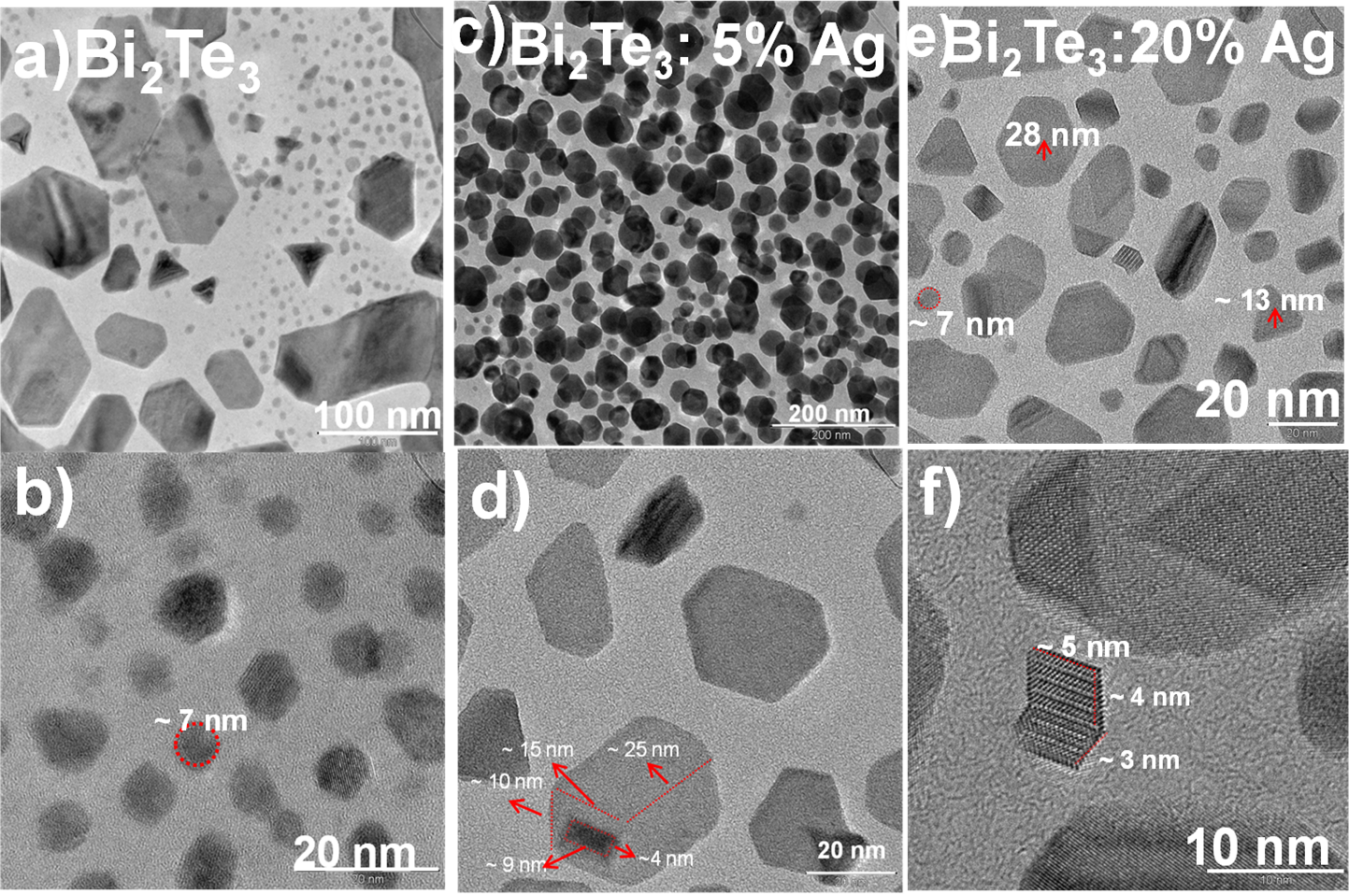
(a) TEM and (b) HRTEM images of Bi_2_Te_3_ with 0% Ag, (c, d) for 5% Ag, (e, f) 20% Ag annealed at 573 K.

Transmission electron microscopy (TEM) and high-resolution TEM (HRTEM) images of Bi_2_Te_3_:Ag samples annealed at 573 K are shown in Figure 3. Figure 3a,b shows the bright-field image and the HRTEM image of the as-prepared Bi_2_Te_3_ samples with no Ag content. There is a wide distribution of particles with various shapes and sizes. To estimate the particles size, the ImageJ software [23] has been used, which yielded an average particles size of the circular nanoparticles of 7–8 nm. The bigger hexagonal shaped particles are of ca. 100 nm and the small triangular and hexagonal nanoparticles are in the range of 40–70 nm.

Figure 3c,d shows the bright-field image and the HRTEM image of Bi_2_Te_3_ samples with 5% Ag annealed at 573 K. Figure 3e,f shows the bright-field image and the HRTEM image of Bi_2_Te_3_ samples with 20% Ag annealed at 573 K with nanoparticles of various shapes and sizes ranging from 7 to 30 nm. In the HRTEM image (Figure 3f) we observed some particles with interesting shapes.

**Figure 4:**
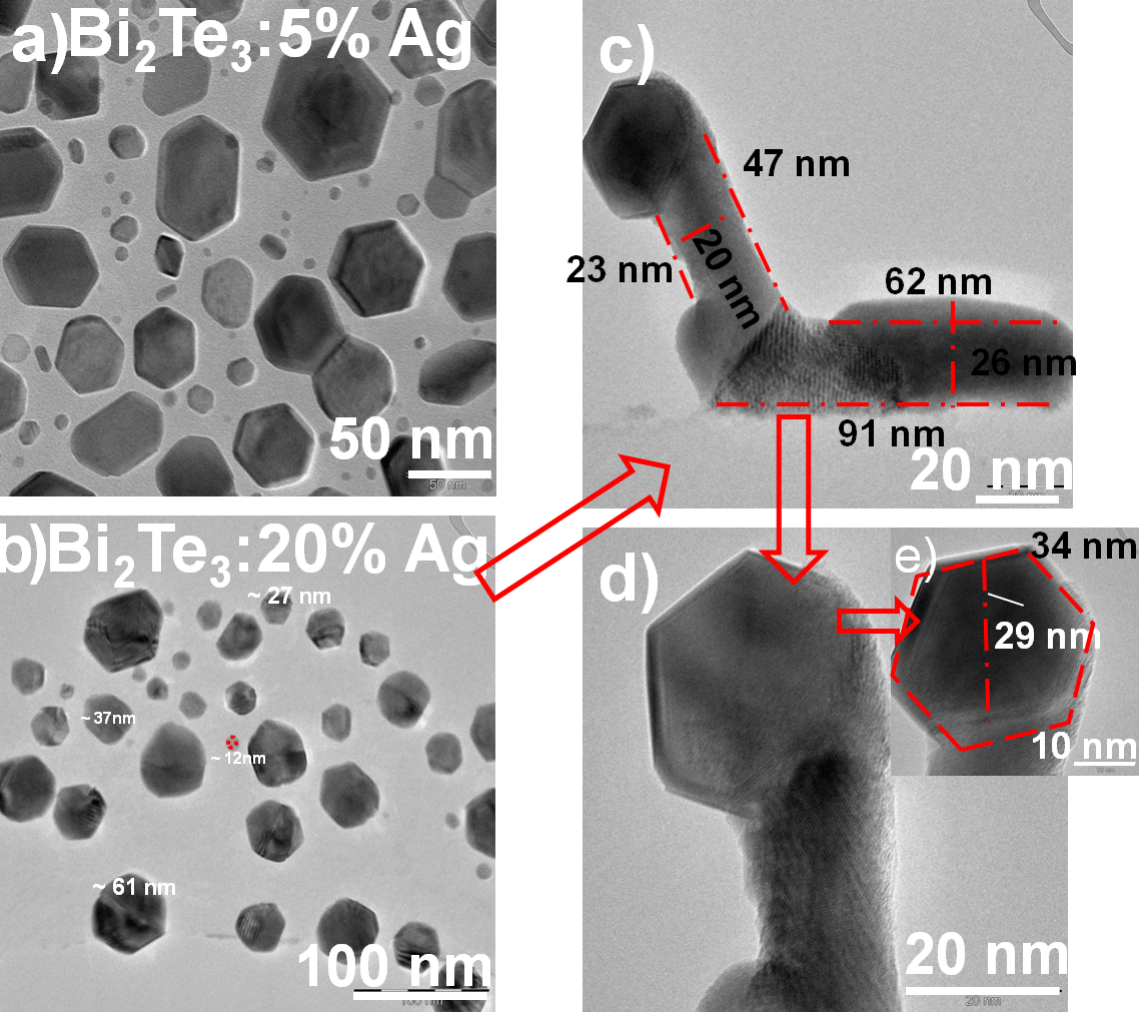
TEM of Bi_2_Te_3_ with (a) 5% and (b) 20% Ag annealed at 773 K, (c, d, e) HRTEM images of Bi_2_Te_3_ with 20% Ag annealed at 773 K.

TEM measurements of Bi_2_Te_3_ with 5% and 20% Ag annealed at 773 K have also been performed as shown in Figure 4a and Figure 4b, respectively. Figure 4a shows the bright-field image and the HRTEM image of the Bi_2_Te_3_ sample 5% Ag annealed at 773 K. One can observe nanoparticles with 3D hexagonal structures along with smaller hexagonal structures which vary from 40 to 80 nm for the large structures and from 2 to 20 nm for the small structures. Figure 4b of Bi_2_Te_3_ with 20 wt % Ag annealed at 773 K shows that particles with mostly hexagonal shaped structures with a wide distribution of sizes from 10 to 60 nm are formed.

In the samples annealed at 573 K there are different shapes, such as tubes, pentagons, triangles, disks and hexagons. Whereas in the samples annealed at 773 K, one can see mainly hexagonal structures. Besides comparison in shapes of the structures size can also be compared in both cases (5%, 20%). In the case of 5% Ag annealed at 773 K, there is a wide distribution in particle sizes from 2 to 20 nm and from 40 to 80 nm, while the particles siazes are 6–25 nm in the sample annealed at 573 K. In the case of 20% Ag a distribution from 10 to 60 nm for samples annealed at 773 K and a distribution from 7 to 30 nm for samples annealed at 573 K have been observed.

In Figure 4c and Figure 4d, a duck-like structure has been observed although it is not present everywhere. It appears to be a combination of hexagonal and tube shaped structures. Its dimensions have been measured, which fit well in the range of particle sizes shown in Figure 4b.

### Thermoelectric measurements

#### Electrical conductivity measurements

**Figure 5:**
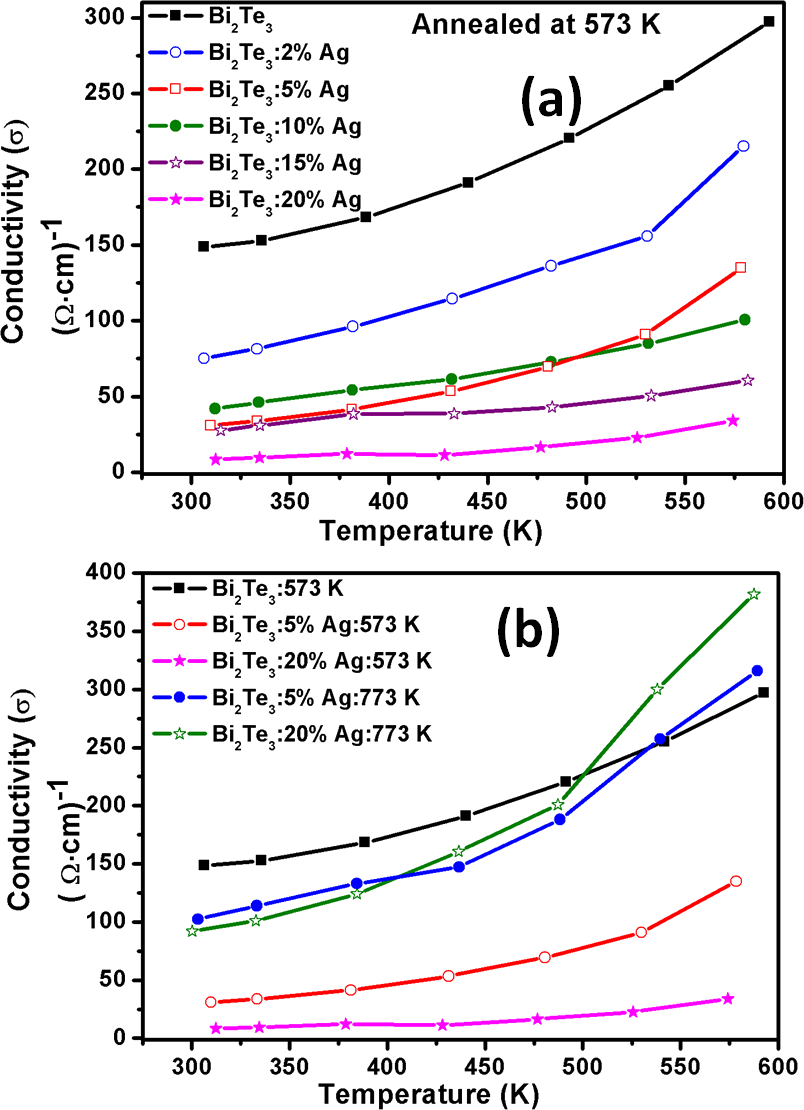
Electrical conductivity (σ) of (a) Bi_2_Te_3_ with (0, 2, 5, 10, 15 and 20%) Ag annealed at 573 K as a function of the temperature; (b) comparison of samples (0, 5, 20%) Ag annealed at 573 K with (5, 20%) Ag annealed at 773 K.

Measurements of the electrical conductivity (σ) in the range of 300–600 K of all samples are shown in Figure 5. The electrical conductivity of all samples increases with increasing temperature as it is expected for a non-degenerated semiconductor. The electrical conductivity of Bi_2_Te_3_ samples with (5, 20 wt %) Ag increases after annealing to higher temperature 773 K in comparison to 573 K annealed samples as shown in Figure 5b.

The electrical conductivity measured at room temperature shows that there is a systematic decrease with increasing Ag content. The highest electrical conductivity at 300 K is ca. 148 (Ω·cm)^−1^ for Ag-free Bi_2_Te_3,_ which decreases by ca. 94% after addition of Ag up to 20% and annealing at 573 K. A decrease by ca. 31% was observed for 5% Ag annealed at 773 K. The conductivity of Ag-free Bi_2_Te_3_ measured at 600 K is ca. 297 (Ω·cm)^−1^, which decreases by ca. 88% for 20% Ag annealed at 573 K.

The decrease in electrical conductivity with increase in Ag content in Bi_2_Te_3_ may be due to the enhanced carrier scattering at the interfaces of metal and semiconductor [20] and due to the presence of oxygen in all samples. An earlier report also suggests that the electrical resistivity of PbTe can increase up to 2–3 orders of magnitude after exposure to air [24]. On the other hand conductivity is higher for samples annealed at 773 K in comparison to 573 K, which may be due to the variation in particle-size distribution. As discussed above, the particle sizes are small in samples annealed at 573 K whereas they are larger in samples annealed at 773 K.

#### Measurement of the Seebeck coefficient (*S*)

**Figure 6:**
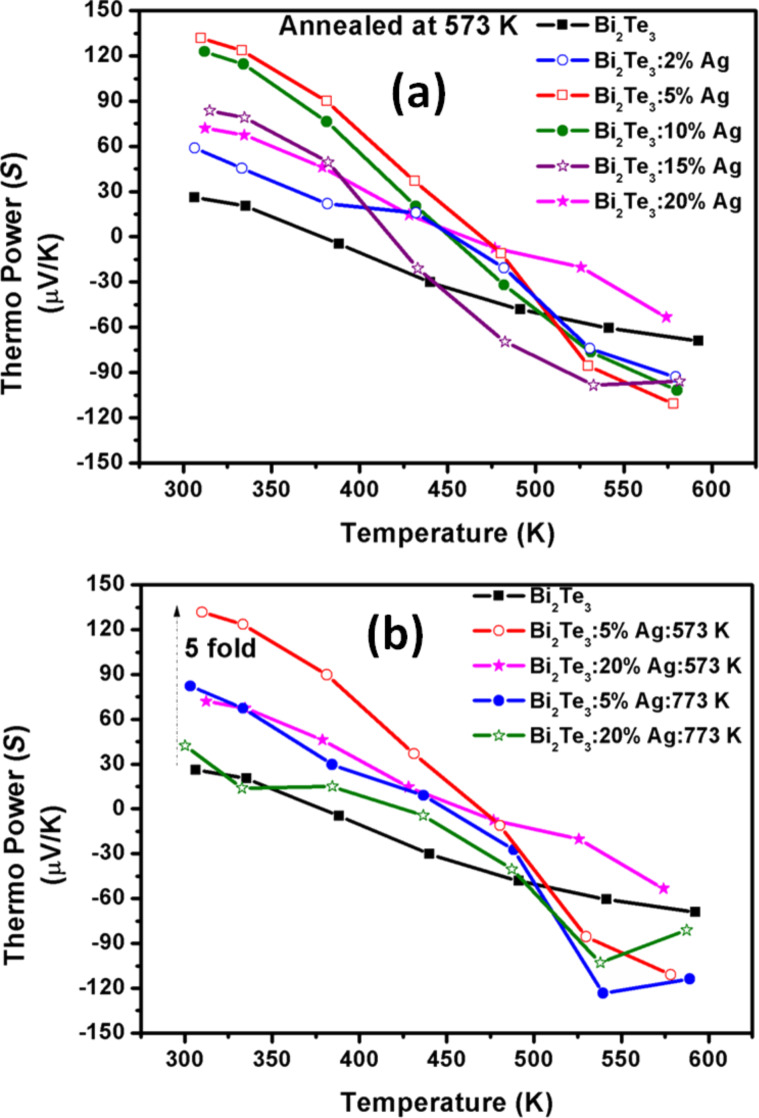
Temperature dependent thermo power measurement (*S*) of (a) Bi_2_Te_3_ with (0, 2, 5, 10, 15, 20%) Ag content annealed at 573 K, and (b) comparison of (0, 5, 20%) annealed at 573 K with (5, 20%) 773 K samples in the temperature range 300–600 K.

Figure 6a shows the Seebeck coefficient as a function of the temperature (300–600 K) of Bi_2_Te_3_ with (0, 2, 5, 10, 15, 20%) Ag content annealed at 573 K. A comparison of Bi_2_Te_3_ samples (0%, 5%, 20% Ag) annealed at 573 K with samples (5, 20% Ag) annealed at 773 K is shown in Figure 6b. All samples exhibit a p-type to n-type conversion with increasing temperature as shown in Table 1 and Figure 6. It can be seen from Table 1, that the transition temperatures vary with different Ag content and the minimum temperature is ca. 378 K for 0% Ag whereas the maximum transition temperature is 469 K for 5% Ag. At room temperature, *S* has a minimum the lowest value of ca. 26 µV/K for Ag-free Bi_2_Te_3_, which increases to a maximum value at 5% Ag. The value decreases again with increasing Ag content after annealing at 573 K. It is interesting to note that all samples show n-type behavior at 600 K. At 600 K, *S* increases with increasing Ag content, *S* ≈ −69 µV/K (0% Ag) which increases up to the maximum value of *S* ≈ −111 µV/K at 5% Ag. At higher Ag contents, there is a decrease to *S* ≈ −101 µV/K for 10%, *S* ≈ −95 µV/K for 15% and *S* ≈ −53 µV/K for 20%. After annealing at 573 K, the thermopower at room temperature for 5% Ag is enhanced by ca. 400% compared to 0% Ag, while only a 2.8-fold increase has been observed for 20% Ag content in Bi_2_Te_3_. At 600 K, there is a 60% enhancement for 5% Ag in comparison to Ag-free Bi_2_Te_3_.

**Table 1:** Comparison of electrical conductivity (σ), Seebeck coefficient (*S*) and power factor (*S*^2^σ) at 300 and 600 K of all samples.

Ag content	Seebeck coefficient (*S*) (µV/K), approximate values		transition temp (K) (at 0 µV/K, p-type to n-type)	conductivity (σ) (Ω·cm)^−1^, approximate values		power factor (*S*^2^σ) (µW·m^−1^·K^−2^) , approximate values	
annealed at 573 K	300 K (p-type)	600 K (n-type)		300 K	600 K	300 K	600 K

0%	26	−69	378	148	297	10	142
2%	59	−93	453	75	215	21	186
5%	131	−111	469	30	135	50	166
10%	122	−101	451	42	100	63	104
15%	83	−95	417	28	61	19	56
20%	72	−53	459	8	34	4	10

annealed at 773 K							

5%	82	−113	450	102	316	69	410
20%	42	−81	425	92	381	16	251

For the samples annealed at 773 K, the Seebeck coefficient of 5% Ag is three-times that of 0% Ag at room temperature. It shows the same enhancement of ca. 60% as 5% Ag annealed at 573 K in comparison to 0% Ag at 600 K. The details are given in Table 1 and Figure 6b.

There is transition of from p-type to n-type behavior with increasing temperature which may be attributed to bipolar diffusion [25–26] and phase transitions [27–28]. In the chalcogenide AgBiSe_2_, there is an exchange of Ag and Bi atoms in the lattice during the phase transition from rhombohedral to cubic that results in the change from p-type to n-type behavior. The exchange of Ag and Bi atoms results in a quasi-metallic state that contributes more conduction electrons, and results in a change from p-type to n-type conduction [27]. A similar work has shown a change in the Seebeck coefficient of AgCuS along with a change in conduction during the phase transition from orthorhombic to hexagonal [28]. In our study, bipolar diffusion with increasing temperature may be responsible for the p–n-type transition. At room temperature, holes are the majority carriers (p-type). When a certain temperature is reached, the *S* becomes zero, because both carrier concentrations become equal (n+, n−). On further increasing the temperature electrons become the majority carriers (n-type) [25]. Another reason for a p–n-type transition is Ge substituting Bi/Te in Bi_2_Te_3_ [26]. Our XRD does not show any phase transition. Hence, we suppose that only bipolar diffusion is the cause for the conduction transition. In order to gain further knowledge about phase transitions and carrier types, further studies such as synchrotron powder X-ray diffraction, heat capacity, Raman spectroscopy, Hall effect and or positron annihilation spectroscopy measurements would be required, which can be the part of a future study.

The enhancement of the Seebeck coefficient can be attributed to carrier filtering. Band bending at the metal–semiconductor interfaces leads to a strong scattering of low-energy electrons whereas high-energy electrons remain unaffected [20–21]. The energy-dependent scattering of electrons is caused by the existence of an electrostatic potential at the interface. The Seebeck coefficient depends on the energy derivative of the relaxation time at the Fermi energy. Thus the electron-energy filtering, in which high-energy electrons remain unaffected, strongly enhances Seebeck coefficient. [20–21,29]. In our samples, one can clearly observe the increased Seebeck coefficient for 5% Ag at the lower annealing temperature, which decreases again for higher Ag content because of the smaller nanoparticles (<30 nm) in the matrix. For the higher annealing temperatures, there is the same trend, but the values of *S* are smaller, which may be attributed to the formation of one type of mainly hexagonal nanoparticles with a wide size distribution as discussed in TEM results.

#### Power factor (*S*^2^σ)

**Figure 7:**
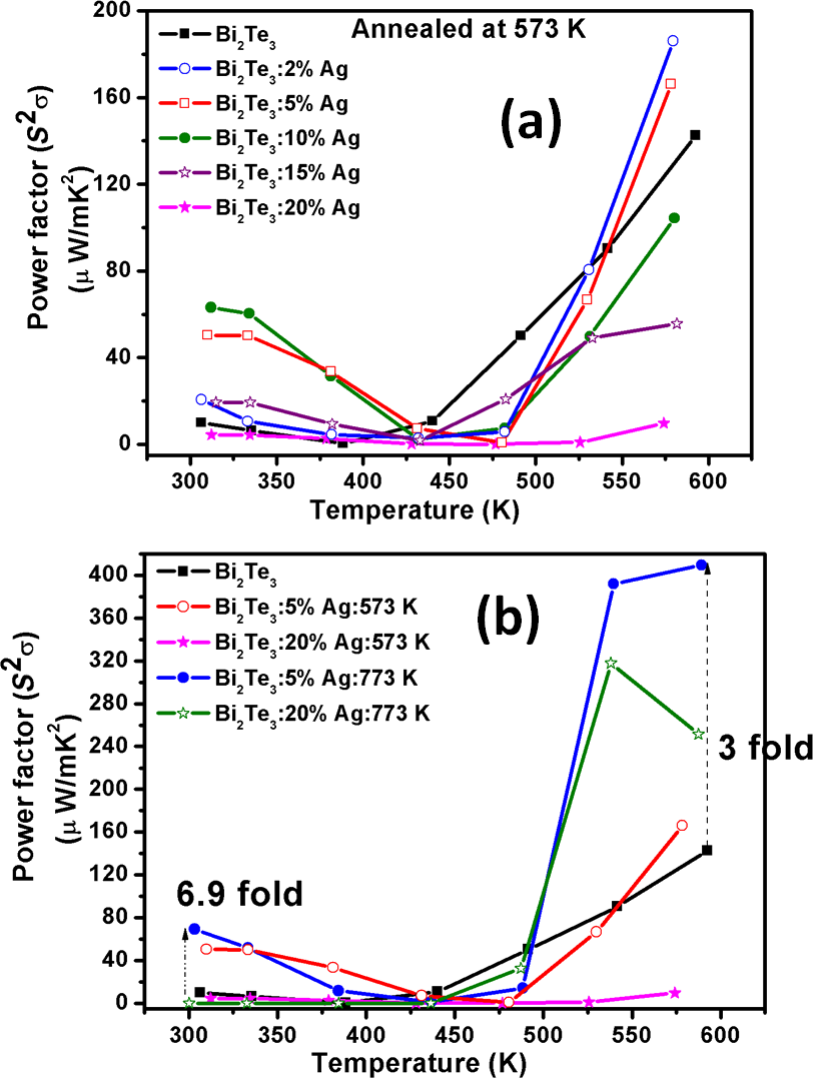
Power factor (*S*^2^σ) as a function of the temperature. (a) Bi_2_Te_3_ with (0, 2, 5, 10, 15, 20%) Ag content annealed at 573 K, and (b) comparison of (0, 5, 20%) Ag content annealed at 573 and 773 K.

Figure 7a,b shows the power factor (*S*^2^σ) in the temperature range 300–600 K (also given in Table 1). It is evident that for Bi_2_Te_3_ annealed at 573 K, the power factor increases with increasing Ag content up to 10% Ag, and decreases again up to 20% Ag at room temperature. At 600 K, the power factor first increases for 2% Ag then it decreased up to 20% Ag. For the samples annealed at 773 K, the power factor increases for 5% Ag at room temperature and the highest value of ca. 410 µW·m^−1^·K^−2^ is observed at 600 K. The power-factor enhancement compared with 0% Ag is ca 6.9-times at 300 K and ca. three-times at 600 K for 5% Ag, annealed at 773 K. For samples annealed at 573 K, the power-factor enhancement is ca. 6.3-times for 10% Ag at 300 K and 1.3-times for 2% Ag at 600 K.

**Figure 8:**
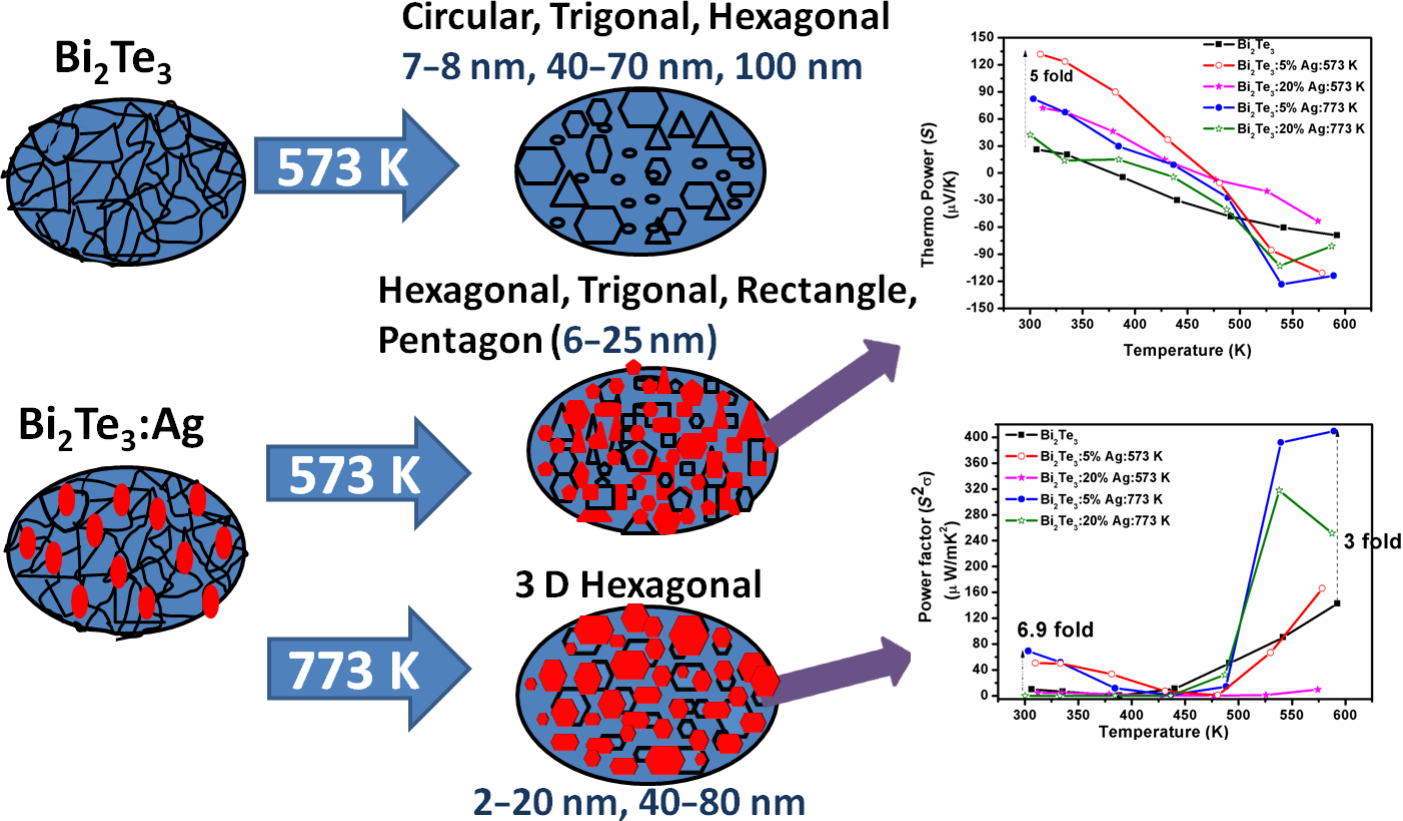
Schematic of effect of Ag nanoparticles in Bi_2_Te_3_ leads to the enhancement in thermoelectric properties.

The nanoparticles in the semiconductor matrix also lead to a reduction of thermal conductivity through phonon scattering at the interfaces, which in turn increases the figure of merit. The present work shows that during the incorporation of Ag in a Bi_2_Te_3_ matrix the distribution of nanoparticles size and shape can be tuned through the annealing temperature, which consequently affects the thermoelectric properties as shown in Figure 8. A five-fold enhancement in thermopower and a 6.9-fold enhancement in power factor for 5% Ag annealed at 573 K and 773 K, respectively, have been achieved in comparison to Ag-free Bi_2_Te_3_.

Our motivation was to focus on low cost and a quick and uncomplicated large-scale production of an efficient nanostructured TE material. In this work, Bi_2_Te_3_ bulk powder and micrometer-sized Ag particles were commercially purchased to avoid variations of intrinsic properties during the synthesis of both Ag and Bi_2_Te_3_. In the present work, the bulk Bi_2_Te_3_ has a Seebeck coefficient of ca. 26 µV/K, which is very low in comparison to synthesized Bi_2_Te_3_ reported in previous reports. However, our main focus is not to compare our results with previous reports, but to study in detail the effect of different Ag contents in Bi_2_Te_3_ and the effect of the annealing temperature on shape and size of the nanoparticles. It can be speculated from the above results that ca. 400% enhancement of *S* and ca. 600% enhancement of *S*^2^σ due to Ag nanoparticles in Bi_2_Te_3_ will also be possible when higher initial values of Seebeck coefficient and power factor for Ag-free Bi_2_Te_3_ are achieved.

## Conclusion

In summary, different sizes and shapes of Ag nanoparticles in different fractions (0–20 wt %) were added to a Bi_2_Te_3_ matrix for a systematic investigation of temperature-dependent thermoelectric properties. Transmission electron microscopy revealed circular, hexagonal, tube-like structures for samples annealed at 573 K, whereas only hexagonal structures were visible for samples annealed at 773 K. The size distribution is also affected by the annealing temperature as smaller size particles (<30 nm) has been found after annealing at 573 K, while together with smaller particles (<30 nm), there are also bigger particles (<80 nm) for samples annealed at 773 K. For the same annealing temperature, a change in Ag content has a a negligible effect on size and shape, whereas a change in annealing temperature leads to change in shape as well as size even for the same content of Ag. For the same content of Ag nanoparticles in Bi_2_Te_3_, the Seebeck coefficient at room temperature increased five-fold after annealing at 573 K, whereas the power factor was enhanced 6.9-fold after annealing at 773 K in comparison to Ag-free Bi_2_Te_3_. This enhancement can be understood on the basis of carrier-filtering. In future, these findings can be helpful to achieve a reduction in thermal conductivity, which in turn will lead to a high figure of merit required for day-to-day thermoelectric applications.
